# Maternal diet-induced hypercholanemia alters gut microbiota and metabolome in adult female Western diet-fed offspring

**DOI:** 10.3389/ebm.2026.10810

**Published:** 2026-01-30

**Authors:** Caroline Ovadia, Saraid McIlvride, Josca M. Schoonejans, Konstantina Spagou, Maria Gómez-Romero, Ann Smith, Georgia Papacleovoulou, Vanya Nikolova, Peter H. Dixon, Elaine Holmes, Julian R. Marchesi, Catherine Williamson

**Affiliations:** 1 Department of Women and Children’s Health, King’s College London, London, United Kingdom; 2 Centre for Reproductive Health, University of Edinburgh, Edinburgh, United Kingdom; 3 Department of Metabolism, Digestion and Reproduction, Imperial College London, London, United Kingdom; 4 School of Health and Social Wellbeing, University West of England, Bristol, United Kingdom

**Keywords:** bile acids, cholic acid, developmental programming, intrahepatic cholestasis of pregnancy, microbiome

## Abstract

Children of mothers with intrahepatic cholestasis of pregnancy (ICP) are more likely to develop metabolic disease later in life. Using a mouse model of gestational cholestasis, we previously found that 18-week-old offspring had metabolic alterations that were exacerbated in female offspring when challenged with a Western diet (WD). Microbiota changes are emerging as a potential mechanism for developmental programming, and the maternal gut microbiota is known to be altered in pregnancy and in ICP. We hypothesized that, in our model, the offspring gut microbiota is altered by maternal gestational disease, potentially impacting future offspring metabolic health. Female mice were fed a cholic acid (CA)-supplemented diet for 1 week preceding and throughout pregnancy to mimic gestational hypercholanemia. Female offspring were challenged with a WD from 12 to 18 weeks of age and cecal contents were collected for metataxonomics and metabolomic profiling. Maternal CA dietary supplementation was associated with markedly increased cecal sulfated bile acid species (up to 387-fold increase). Whilst WD-feeding of offspring was associated with a greater proportion of primary to secondary bile acids, and more tauro-conjugated bile acids than for offspring fed a normal diet, this adaptation to WD-feeding was not evident for those whose mothers were fed a CA-supplemented diet. Indeed, WD-fed offspring of CA-supplemented mothers had a >2-fold reduction in CA and dehydrocholic acid levels compared to those from NC-fed mothers. This corresponded with an altered profile of cecal microbiota, with clear separation of microbiotal profiles according to maternal diet in the WD-fed, but not NC-fed, offspring. This observational mouse study has shown that exposure to maternal hypercholanemia can significantly impact the effects of an obesogenic diet on offspring intestinal bile acid metabolism and gut microbiota, likely increasing their vulnerability to metabolic dysfunction when exposed to the “second hit” of an unhealthy postnatal environment.

## Impact statement

The gut microbiota in gestational disease and as a means of developmental programming remains understudied. As data emerge about the contribution of the gut microbiota to metabolic health, studying maternal and offspring microbiota in the context of maternal metabolic disease becomes increasingly important. Whilst the impact of the maternal environment on early offspring microbiotal composition is more evident, understanding whether this directly impacts the longer-term intestinal microbiota is less clear. Herein, we report changes in the microbial communities and bile metabolome for both maternal and offspring intestine following maternal cholic acid feeding. Exposure to maternal hypercholanemia also altered metabolic responses to postnatal overnutrition in female offspring, potentially predisposing them to future metabolic disease. This work illustrates the need for follow-up studies looking at the impact of maternal bile acids on offspring gut microbiota. Our findings provide novel information on how maternal metabolic diseases (including cholestasis) may lead to adverse effects in their offspring, and could provide a steppingstone to the development of new therapies targeting the intestinal bile acid system and/or the gut microbiota.

## Introduction

As pregnancy advances, there is a shift in maternal metabolism that results in elevated serum bile acids (hypercholanemia), dyslipidemia and relative insulin resistance to support fetal growth [1]. In some pregnant women, serum bile acid concentrations become pathologically raised and intrahepatic cholestasis of pregnancy (ICP) develops. ICP is the most common liver-specific disease of pregnancy, estimated to affect approximately 0.3–5.6% of pregnancies, depending on ethnicity and geography [2]. As well as hypercholanemia, women with ICP have pruritus, raised liver enzymes and increased risks of preterm labor, meconium-stained amniotic fluid, and stillbirth [3]. Women with ICP are also at higher risk of developing gestational diabetes mellitus [4]. In addition to risks to fetal health, we have previously demonstrated an increased risk for both humans and mouse offspring to develop metabolic disease later in life [5]. Female sixteen-year-old children of ICP mothers were found to have reduced serum high density lipoprotein (HDL)-cholesterol and increased waist and hip girth, while males had increased fasting insulin and a higher body mass index (BMI), relative to controls [5]. In a mouse model of cholestatic pregnancy, 18-week-old offspring had increased hepatic free fatty acids, with females also showing mild hepatosteatosis and increased inflammatory markers [5]. When challenged with a Western diet, female offspring developed a more severe obesogenic and diabetic phenotype when compared to females from non-hypercholanemic mothers [5]. Epigenetic changes could be responsible for this metabolic programming, as we showed that hypercholanemia in pregnancy caused alterations in the offspring DNA methylation. However, recent studies have shown that changes to the microbiota are another potential route for transmission of disease risk [6, 7].

The initial gut microbiota is mainly acquired at birth. Vertical transmission of microbes from mother to infant has been shown to be integral to the development of infant microbiota, with the maternal gut being a major source of colonizing bacteria in the infant intestine [8]. Mode of birth has been shown to strongly influence newborns’ intestinal microbiota [9–11]. We have demonstrated in mice that normal pregnancy is associated with changes to the gut microbiome, enhancing the abundance of *Bacteroidetes* and sulfur-utilizing bacteria, with an associated increase in microbial richness and diversity [12]. A similar effect was found in mice fed a diet supplemented with cholic acid (CA) [12]. ICP further impacts the microbiota, with significant differences in the relative abundance of several bacteria found between ICP patients and healthy pregnant controls [13–15], which was further altered by treatment with ursodeoxycholic acid [16]. Therefore, we hypothesized that, like their mothers, offspring of mothers with ICP have altered gut microbiota, which contributes to their increased susceptibility to developing metabolic disease. The cecum is a key intestinal organ involved in bacterial-mediated short-chain fatty acid production and thought to act as a bacterial reservoir for the colon [17]. We used a murine model of CA-supplemented diet before and during pregnancy, which mimics the hypercholanemia in ICP by increasing maternal serum bile acids [18], to assess the impact upon cecal microbiota and bile acid and lipid profiles in offspring fed a normal chow or a Western diet.

## Materials and methods

### Animal studies

**FIGURE 1:**
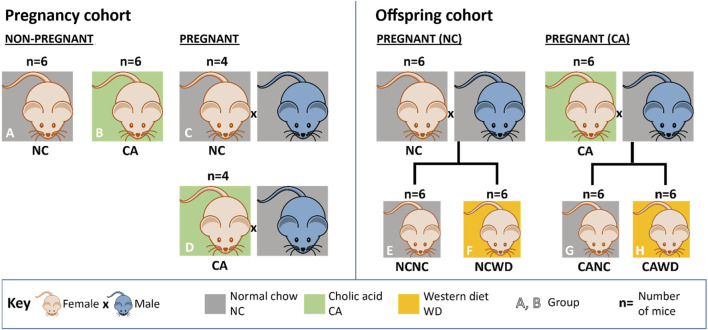
Experimental design. Numbers for non-pregnant females (A,B) and pregnant females (C,D) in the pregnancy cohort; and numbers for dams (not used for this study) generating female offspring (E–H) in the offspring cohort. (A) non-pregnant females fed on normal chow (NC). (B) non-pregnant females fed on 0.5% cholic acid-supplemented chow (CA). (C) pregnant NC-fed dams. (D) pregnant CA-fed dams. (E,F) female NCNC, NCWD, CANC and CAWD offspring. Dams for the offspring cohort were subjected to the same treatment regimen as dams in the pregnancy cohort, except were left to litter. CA = cholic acid-supplemented diet. CANC = NC-fed offspring of CA-fed dams. CAWD = WD-fed offspring of CA-fed dams. NC = normal chow. NCNC = NC-fed offspring of NC-fed dams. NCWD = WD-fed offspring of NC-fed dams. WD = Western diet.

All experimental procedures were performed in accordance with the UK Animals (Scientific Procedures) Act of 1986 and approved by the Ethical Committee for Animal Welfare at Imperial College London and the UK Home Office (70/6867). Experimental procedures have previously been described [5]. Briefly, mice were housed within barrier facilities, with a 12-h light/dark cycle and free access to food and water. Female C57Bl/6J mice (10–12 weeks old, Harlan, UK now Envigo, UK) that had previously delivered one litter were used. Female mice were randomly allocated to receive normal chow diet (NC; RM3 diet, Special Diets Services, UK) or RM3 diet supplemented with 0.5% cholic acid (CA; LBS Biotechnology, UK). Dams were fed the experimental diet for 1 week prior to mating with C57BL/6J males (also fed NC, RM3 diet) and continued the same diet throughout gestation. Dams were either euthanized on the 18th day of gestation for collection of maternal samples (pregnancy cohort) or after weaning of their litters (offspring cohort). Non-pregnant females were culled after 18 days on their respective diets to match the duration of CA-feeding in the pregnancy cohort (Figure 1). After delivery, litters were reduced to 2 females and 2 males per dam and the CA-supplemented diet was removed. Pups were weaned at 3 weeks old and fed NC until 12 weeks, after which 1 female offspring from each litter was fed an obesogenic ‘Western diet’ (WD; LBS Biotechnology, UK) while littermates remained on NC diet. Body weight and food intake were monitored weekly; these data were previously published [5]. Offspring were euthanized at 18 weeks by carbon dioxide inhalation after a 4-h fast, and ceca including contents were collected and snap frozen in liquid nitrogen. While frozen, ceca were longitudinally divided, and intestinal tissue was dissected from cecal contents. In total, 4-6 mice per group were available for analysis (n = 6 per group for non-pregnant mice and female offspring of NC- and CA-fed mothers who were themselves fed NC or WD; and n = 4 per group for pregnant mice; Figure 1). Only female offspring were studied in this instance as these were found to have the most severe metabolic phenotype in our previous work [5].

### Metabolic profiling

#### Sample preparation

Aqueous extraction was performed for cecal content samples using a protocol adapted from Want and co-workers [19] as previously described [12]. Briefly, 1 mm zirconium beads and 1.2 mL of chilled methanol/water (1:1) solution were added to the samples (50 mg) and homogenized using a bead beater (Bertin Technologies; 6500Hz for 40s). Blank samples were also prepared. Samples were centrifuged (5417R, Eppendorf, Germany) at 3,486 × g for 20 min at 4 °C and the supernatant aliquoted into fresh microcentrifuge tubes. Aliquots were dried using a vacuum concentrator (Eppendorf Concentrator Plus) for 3 h at 45 °C in V-AQ mode and stored at −40 °C until analysis.

Organic extraction was performed on the solid precipitate from the aqueous extraction. An aliquot (1.2 mL) of chilled dichloromethane/methanol solution (3:1) was added to the pellet, frozen on dry ice, and reloaded into the bead beater as above. Following centrifugation at 3,486 × g at 4 °C for 20 min, two aliquots of 400 μL organic phase supernatant were transferred to glass vials for lipid and bile acid ultra-performance liquid chromatography-tandem mass spectrometry (UHPLC-MS/MS) profiling. Samples were evaporated at room temperature in an extractor hood overnight and stored at −40 °C until analysis.

#### Reversed-phase (RP)-UHPLC-MS lipid profiling of organic extracts

The protocol used here was adapted from Vorkas [20] and Spagou [21]. Organic extracts were reconstituted in an isopropanol/acetonitrile/water mixture (2:1:1, 250 µL) and vortexed for 30 s, sonicated for 5 min, vortexed again for 30 s, and centrifuged at 3,486 × g for 30 min at 4 °C. Supernatant was transferred into glass inserts in LC-MS vials. A quality control (QC) sample was also prepared by pooling 50 µL of each sample to assess analytical reproducibility. Lipid profiling was carried out on an Acquity UPLC system (Waters Corp, USA) coupled to a XEVO G2 QTOF Mass Spectrometer (Waters MS Technologies, UK). Chromatography was performed using an Acquity UPLC CSH C18 column (1.7 µm, 2.1 mm × 100 mm; Waters Corporation, USA), as previously described [12]. Mass spectrometry was performed using both positive and negative electrospray ionization (ESI) modes, as previously described [12].

#### RP-UHPLC-MS bile acid profiling of combined aqueous and organic extracts

The protocol was adapted from Sarafian and colleagues [22]. Aqueous extracts were reconstituted in a propanol/water mixture (1:1, 150 µL). The supernatant was used to reconstitute the organic extracts. Following centrifugation at 3,486 x g for 30 min at 4 °C, the supernatant (100 µL) was transferred into glass inserts in LC-MS vials. A QC sample was prepared by pooling 40 µL of each sample. UHPLC-MS analysis of bile acids was performed using an Acquity UPLC system (Waters Corp, USA) coupled to a XEVO G2 QTOF Mass Spectrometer (Waters MS Technologies, UK). Separation was performed in an ACQUITY BEH C8 column (1.7 μm, 100 mm × 2.1 mm) and the mass spectrometer operated in ESI negative ion mode, under the same conditions as described previously [12].

#### Data pre-processing of bile acid/lipid data

The DataBridge tool implemented in MassLynx software (Waters Corporation, Milford, MA) was used to convert MS raw data to netCDF format. The XCMS package in R was used to process the data, giving pairs of m/z_RT (mass to charge ratio_retention time) and intensity values of detected metabolite features in each sample [23]. The data were normalized to total area normalization. Orthogonal projection to latent structures discriminant analysis (OPLS-DA) were employed to analyze UHPLC-MS data in a multivariate setting, using the SIMCA package (v.13.0.2; Umetrics, Umea, Sweden). Prior to model fitting, features underwent Pareto scaling to minimize the importance of large values.

#### Metabolite assignment

Chromatographic peaks were annotated as metabolites by matching m/z measurements to theoretical values from in-house databases and online databases (Human metabolite database (HMDB);^1^), Kyoto Encyclopedia of Genes and Genomes (KEGG;^2^), LIPID MAPS^3^, and METLIN^4^. Tandem MS fragmentation patterns provided further structural information. Metabolite assignment was confirmed by comparing retention times and MS/MS data against commercially available standards.

#### Materials

All LC-MS grade solvents (acetonitrile, isopropanol) and additives (ammonium formate, formic acid) to prepare the mobile phases were purchased from Sigma-Aldrich (UK), apart from the water which was from Fisher (Germany). Methanol and dichloromethane used in sample preparation were from Sigma-Aldrich (UK). Bile acid and lipid standards were obtained from Steraloids (Newport, RI, US) and Avanti Polar Lipids, respectively (Alabaster, AL, US).

### 16S ribosomal RNA (rRNA) gene sequencing

Cecal content samples were homogenized using a bead beater, and DNA was extracted using QIAamp DNA Stool Mini kit (Qiagen, UK), according to the manufacturer’s protocol. Metataxonomic sequencing was performed by Research and Testing Laboratories (Lubbock, Texas). 16S rRNA sequencing was carried out using the Roche 454, using V1-V2 primers (28F and 338R) according to published protocols [24]. The extracted DNA was sequenced, and 117,875 reads were generated and after filtering this figure was reduced to 111,016 reads; the median read depth after filtering was 1,475 per sample with a range of 473–6568 reads. Reads were not subsampled and were center log ratio transformed before being analyzed in R. Data analysis was carried out using Mothur (v1.25.0); following the 454 SOP Pipeline [25]. Sequences were aligned using the Silva bacterial database (https://www.arb-silva.de/SILVA 115), and the Wang method was used to classify sequences according to the Ribosomal Database Project reference sequence files (reference = trainset9_032012.rdp.fasta, taxonomy = trainset9_032012.rdp.tax) [26]. Nonmetric multidimensional scaling (NMDS) plots were produced using the UniFrac weighted distance matrix created by Mothur, and analysis was carried out using the Vegan library within R [23]. Alpha diversity indices (Shannon, Chao 1 and Observed OTU) were calculated using Mothur, and compared using R, GraphPad Prism and STAMP statistical software [27]. Heatmaps were produced in R (Version 4.2.0) using packages Rcpp, NMF, RColorBrewer, ggplot2, vegan and gplots.

### Statistical analysis

Fold changes in cecal metabolites were calculated by dividing the data from the experimental group by the control group, for the comparisons shown. Fold change values less than one were replaced by the negative of their inverse. Comparison of cecal metabolites between two groups was performed on SIMCA output: *p*-values were calculated by two-tailed Student’s t-test assuming unequal variance and coefficient of variation percentage in Microsoft Office Excel (Redmond, WA), and adjusted for multiple comparisons using the Benjamini-Hochberg method in R.

When comparing cecal bile acid data (e.g., for primary/secondary ratio, sulfation and T-conjugation rates) or microbial diversity data for more than two groups, two-way ANOVA (dams) or repeated measures mixed modelling (female offspring, accounting for sibling effects) followed by Fisher’s LSD test for multiple comparisons was carried out in GraphPad Prism. *P*-values of less than 0.05 were considered significant.

## Results

### The offspring cecal metabolome profile is affected by maternal cholic acid dietary supplementation, predominantly affecting the bile acid composition

Cecal contents were analyzed by untargeted UPLC-MS to identify changes in the metabolic profile, specifically for bile acids and lipids, due to maternal and offspring diet. No differences were observed in the cecal metabolome between adult female offspring from NC- or CA-fed mothers, when offspring received NC (Table 1). However, in female offspring challenged with a WD, there was a significant difference in the overall cecal bile acid profile between female offspring from NC- and CA-fed mothers (Table 1). Irrespective of maternal diet, a WD caused significant changes in the overall metabolome when compared to NC in female offspring.

**TABLE 1:** Cecal metabolome.

Offspring diet	Groups	Maternal diet	Sex	Bile acids	Lipids	
				ESI-	ESI+	ESI-
Normal	Offspring	Normal vs. cholic acid	F	NS	NS	NS
Western	Offspring	Normal vs. cholic acid	F	**0.036**	NS	NS

Summary of Orthogonal Projections to Latent Structures Discriminant Analysis (OPLS-DA) models. ESI-, electrospray ionization in negative mode; ESI+, electrospray ionization in positive mode; F, female; NS, not significant. P-values determined by Student’s t-test following Pareto scaling and model fitting with SIMCA. Significant changes (p<0.05) are shown in bold. Female offspring, n = 6 per group.

### Maternal cholic acid-feeding alters the cecal bile acid profile of female offspring fed a Western diet, reducing the proportion of primary, and taurine-conjugated, bile acid species

**FIGURE 2:**
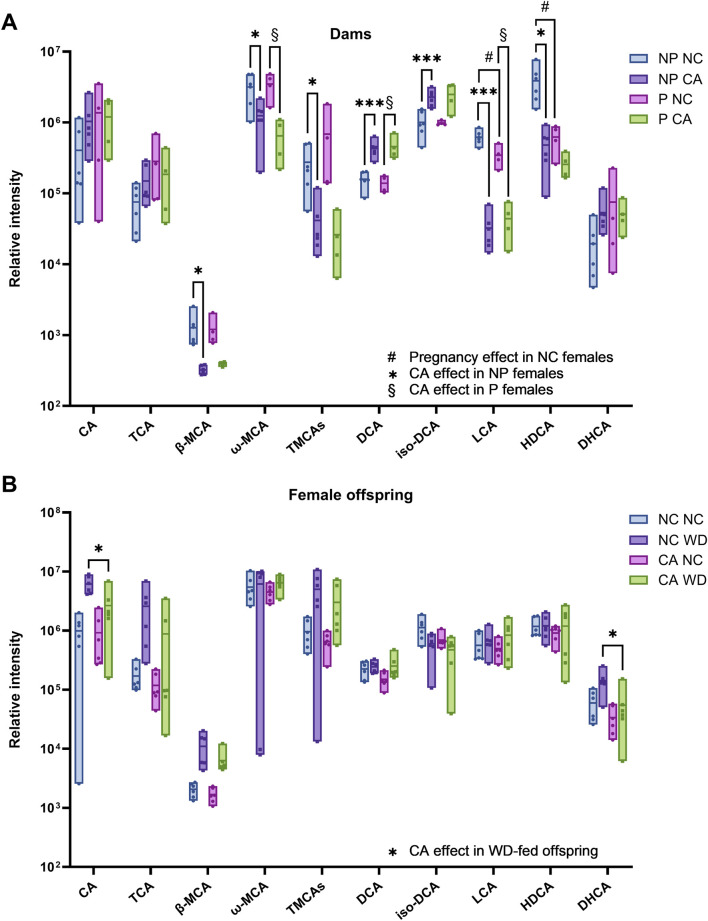
Individual bile acid species present in cecal content determined by bile acid profiling UHPLC-MS/MS. **(A)** Pregnant (P) and non-pregnant (NP) mice fed normal chow (NC) or cholic acid (CA)-supplemented diet. *p < 0.05, **p < 0.01, ***p < 0.001 as determined by Student’s t-test following Pareto scaling and model fitting with SIMCA (full statistical information shown in Supplementary Table S1) comparing two groups to test the following effects: ^#^Pregnancy effect in NC-fed females (P NC vs. NP NC); *CA effect in non-pregnant females (NP CA vs. NP NC); ^§^CA effect in pregnant female (P CA versus P NC). n = 4–6. **(B)** Female offspring from NC- or CA-fed dams challenged with Western diet (WD) or NC. *p < 0.05 as determined by Student’s t-test following Pareto scaling and model fitting with SIMCA (full statistical information shown in Supplementary Table S2. n = 6. Box plots show mean and range. Abbreviations: CA, cholic acid; TCA, taurocholic acid; β-MCA, β-muricholic acid; ω-MCA, ω-muricholic acid; TMCAs, Tauro-muricholic acids; DCA, deoxycholic acid; LCA, lithocholic acid; HDCA, hyodeoxycholic acid; DHCA, dehydrocholic acid.

Based on the findings of these models, individual bile acid species were examined in the cecal contents of dams (referring to pregnant and non-pregnant females) and of female offspring. Unsurprisingly, dietary CA supplementation significantly impacted the overall cecal bile acid profile in both pregnant and non-pregnant females (Figure 2A; Supplementary Table S1), confirming findings in previous studies [12]. In particular, there were large increases in sulfated bile acids in those fed CA compared to NC, up to a 387-fold increase, which could be a potential mechanism for eliminating excess dietary bile acids in these groups; this was contrasted by an overall reduction in taurine-conjugated bile acids with CA supplementation (p < 0.001) (Figure 2B; Supplementary Table S1). Consistent with the OPLS-DA model findings, when investigating the effect of maternal CA-feeding in females given WD, there was more than a two-fold reduction in CA (p = 0.02) and DHCA (p = 0.03) in CA WD females compared to NC WD females (Figure 2B; Supplementary Table S2). There was no difference in cecal bile acid species in response to maternal CA-feeding in NC-fed offspring (Supplementary Table S2).

**FIGURE 3:**
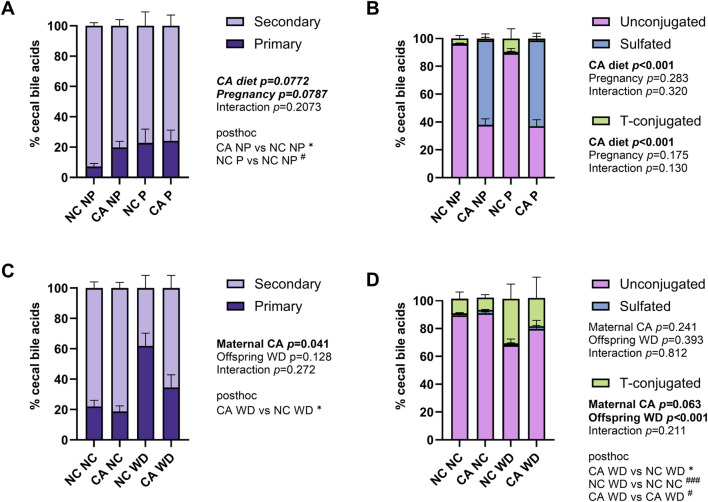
Summary of cecal bile acid profile. **(A,C)** Distribution of cecal bile acids by conjugation in the cecal content in dams **(A)** and female offspring **(C)**. Unconjugated: CA, DCA, iso-DCA, βMCA, ωMCA, LCA, HDCA, DHCA. Taurine-conjugated: TCA, TMCAs. Sulfated: six unspecified BAs. **(B,D)** Distribution of primary and secondary cecal bile acids in dams **(B)** and female offspring **(D)**. Primary: CA, TCA, βMCA, TMCAs. Secondary: DCA, iso-DCA, HDCA, LCA, DHCA, ωMCA. *P*-values reflect outcomes of two-way ANOVA (dam data) or repeated measures mixed effect modelling (female offspring, with dam ID as the repeated measures factor to control for sibling effects) with Fischer’s LSD multiple comparisons using log-transformed data: **(A,C)** the ratio between primary and secondary bile acids; **(B,D)** the percentage of bile acids that were sulfated or T-conjugated. *p < 0.05 for maternal CA diet effect; ^#^ *p* < 0.05, ^###^ *p* < 0.001 for pregnancy (dams) or offspring WD effect (female offspring). Abbreviations: CA, cholic acid; TCA, taurocholic acid; β-MCA, β-muricholic acid; ω-MCA, ω-muricholic acid; TMCAs, tauro-muricholic acids; DCA, deoxycholic acid; LCA, lithocholic acid; HDCA, hyodeoxycholic acid; DHCA, dehydrocholic acid; NC, normal chow diet; NP, not pregnant; P, pregnant; WD, Western diet.

Individual bile acid species were grouped to show broad changes in bile acid composition. The majority of the cecal bile acid pool consisted of secondary bile acids in both dams and offspring (Figures 3A,C). Primary bile acids tended to be increased in pregnancy and when dams were fed a CA-supplemented diet (p = 0.079 and p = 0.077, respectively) (Figure 3A). Sulfation rates were more than twice as high in CA-fed females (p < 0.001) (Figure 3B). Offspring from NC-fed dams fed a WD had the highest proportion of primary to secondary bile acids (Figure 3C), which is commonly seen in human obesity [28]; this was in contrast to the CA WD mice, who had proportionately greater secondary BAs than the NC WD mice, consistent with higher proportions of unconjugated BAs, indicative of the need for bacterial deconjugation prior to modification to secondary BAs in the intestine (Figure 3D).

### Maternal cholic acid dietary supplementation did not impact the cecal lipid profile, in offspring fed either normal chow or Western diets

Due to the role of the cecal microbiota in short-chain fatty acid production and the changes in lipid metabolism previously observed in the serum and liver of these mice, the lipid profile of cecal contents was measured using lipid profiling UHPLC-MS/MS. Consistent with the overall model (Table 1), no individual lipid was demonstrated to differ in the cecal content of offspring from mothers fed different diets (NC or CA) (Table 2).

**TABLE 2:** Lipid species in female offspring cecal content.

Metabolite (molecular species level)	NC NC vs. CA NC		NC WD vs. CA WD	
	Fold change	p-Value	Fold change	p-Value
PC(16:0/18:1)	−1.68	NS	−1.53	NS
PC(16:0/18:2)	−1.72	NS	−1.85	NS
PC(16:0/20:4)	−1.82	NS	−1.94	NS
PC(36:2)	−3.61	NS	−2.21	NS
PC(36:2)	−2.67	NS	−2.34	NS
LPC(16:0)	1.00	NS	−1.97	NS
LPC(18:0)	1.07	NS	−1.80	NS
TG(16:1/18:1/18:2)	−1.26	NS	−1.28	NS
TG(18:1/18:2/18:2)	−1.08	NS	−1.04	NS
TG(18:1/18:2/18:3)	1.05	NS	−1.26	NS
TG(12:0/18:1/18:1) and TG(14:0/16:1/18:1) and TG(14:0/16:0/18:2) and TG(14:1/16:0/18:1) and TG(16:1/16:1/16:0)	−2.01	NS	−1.05	NS
TG(14:0/16:0/18:1) and TG(16:0/16:0/16:1)	−1.52	NS	1.08	NS
TG(16:0/16:1/18:1) and TG(14:0/18:1/18:1)	−1.47	NS	1.09	NS
TG(16:0/18:1/18:1)	−1.33	NS	1.05	NS

Table showing lipid species present in cecal contents as determined by lipid profiling UHPLC-MS/MS, in female offspring. Offspring from normal chow (NC)-fed mothers, or cholic acid (CA)-fed mothers, challenged with Western diet (WD) compared to NC., Fold change between dietary groups shown. n = 6 per group. TG, triglyceride; PC, phosphatidylcholine; LPC, lysophosphatidylcholine. *P*-values determined by Student’s t-test following Pareto scaling and model fitting with SIMCA. NS, not significant.

### Maternal cholic acid supplementation is associated with separately-clustered profiles of cecal microbiota in offspring fed a Western diet

**FIGURE 4:**
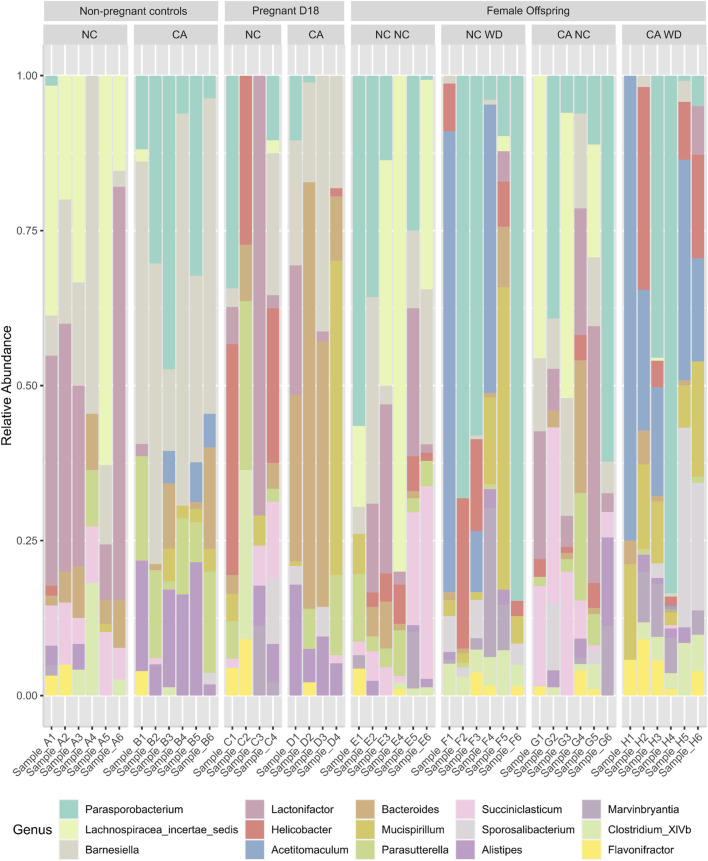
Bacterial genera present in cecal contents. Stacked bar chart representing the relative abundance of the top 15 bacterial genera in different maternal and offspring dietary groups. D18, gestational day 18; NC, normal chow; CA, cholic acid supplemented diet; NC NC, NC-fed offspring from NC-fed mothers; NC WD, WD-fed offspring from NC-fed mothers; CA NC, NC-fed offspring from CA-fed mothers; CA WD, WD-fed offspring from CA-fed mothers.

**FIGURE 5:**
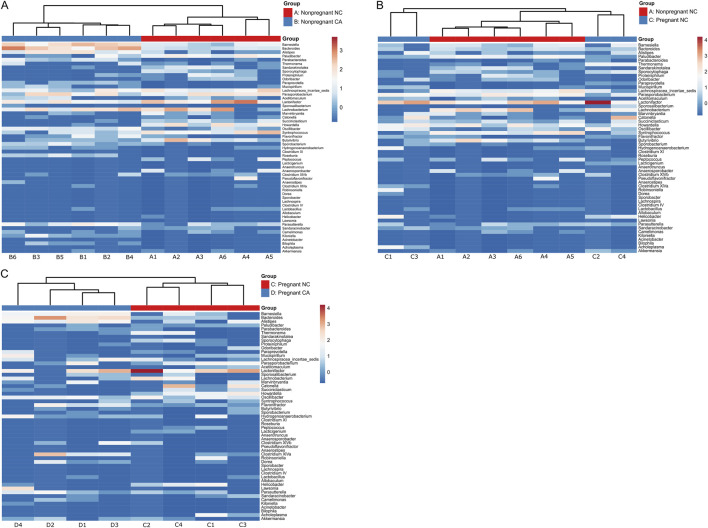
Taxonomic analysis of maternal cecal microbiome. Heat map of unsupervised cluster analysis showing bacterial community composition of cecal contents of non-pregnant and pregnant mice at genus level. **(A)** Non-pregnant normal chow (red) and non-pregnant cholic acid fed (blue). **(B)** Non-pregnant (red) and pregnant (blue) normal chow fed. **(C)** Pregnant normal chow (red) and pregnant cholic acid fed (blue). n = 4-6 per group. Letters and numbers refer to experimental groups as shown in Figure 1 (e.g., Sample A1 = sample from a non-pregnant chow-fed adult female).

Due to the reciprocal relationship between bile acids and microbiota in the gut, we next analyzed the cecal microbiota. Figure 4 shows relative abundance of the top 15 most abundant bacterial genera present in the cecal contents. Mice supplemented with CA had a microbiota in which Bacteroidetes (such as *Barnesiella* and *Bacteroides*) predominated, whilst those fed NC or WD had proportionately more Firmicutes (including *Parasporobacterium* and *Lachnospiraceae*). In non-pregnant mice, unsupervised cluster analysis showed a clear distinction between NC-fed and CA-fed mice with regards the overall composition of the cecal microbiota (Figure 5A). When non-pregnant NC-fed mice were compared to pregnant NC-fed mice, the pregnant mice clustered into two different groups (Figure 5B). This clustering is consistent with our previous study, which showed increased diversity of bacteria in pregnancy [12]. Finally, similar to non-pregnant mice, diet supplementation with CA in pregnant mice results in a clear separation from NC-fed mice (Figure 5C).

**FIGURE 6:**
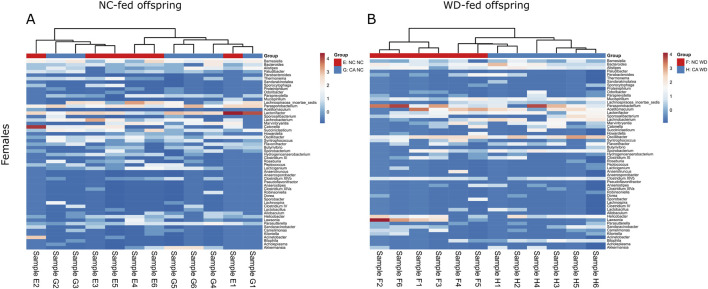
Taxonomic analysis of offspring cecal microbiome. Heat map of unsupervised cluster analysis showing bacterial community composition of cecal contents from female offspring. Showing the top 55 bacterial genera. **(A)** Normal chow-fed females from normal chow-fed mothers (NC NC; red) and CA-fed mothers (CA NC; blue). **(B)** Western diet-fed females from normal chow-fed mothers (NC WD; red) and CA-fed mothers (CA WD; blue). Letters and numbers refer to experimental groups as shown in Figure 1 (e.g., Sample E1 = sample from one NC NC female offspring).

**FIGURE 7:**
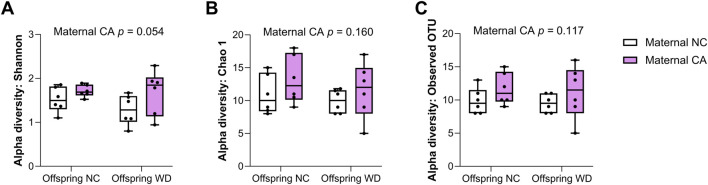
Alpha diversity of cecal contents. Alpha-diversity indices **(A)** Shannon, **(B)** Chao 1 and **(C)** Observed OTUs in female offspring. NC, normal chow; CA, cholic-acid supplemented diet; WD, Western diet. *P*-values determined by repeated measures two-way mixed effects modelling for the effect of maternal CA diet, offspring WD, and the interaction between them; with dam ID as the repeated measure to control for sibling effects. n = 6 per group.

In the female offspring, the heatmap revealed a distinct pattern in the distribution of microbiota in WD-fed mice; there was clear segregation between samples from CA-fed mothers and NC-fed mothers, although two outliers are evident in the NC WD group (Figure 6B). This segregation is not seen in the NC-fed offspring, showing that WD is required to unmask this effect (Figure 6A). No significant changes were observed in Chao1 index or in observed OTUs in female offspring, but the Shannon index tended to increase following exposure to maternal CA-feeding, suggestive of an increased microbial diversity (p = 0.054) (Figure 7).

## Discussion

We demonstrated that offspring cecal metabolome and microbiome are influenced by the maternal diet, with metabolomic differences revealed when offspring are challenged with a Western diet. By assessing the impact for adult offspring, we demonstrated that gestational alterations in the maternal environment can persist far beyond the neonatal period, and influence the response to a later metabolic challenge. That an altered offspring microbiota was not evident according to maternal diet (CA-supplementation or NC feeding) when the offspring were fed the same, NC, diet, suggested that these impacts are less associated with a direct maternal-neonatal transfer of differing microbiota, and more that early programming of the offspring influences later response to Western diet feeding at the intestine.

Developmental programming of disease is a well described phenomenon [29, 30], and an altered microbiota is emerging as a potential route for the transmission of disease susceptibility from one generation to the next [31]. There is evidence that disruption of early life microbiota is linked to long-term health disorders such as obesity, inflammatory bowel disease and atopic disease [32–35]. Several studies have reported changes in the neonatal gut microbiota in infants of mothers with obesity or gestational diabetes mellitus [31], but there are few studies examining the impact of maternal hypercholanemia or cholestasis on the offspring microbiota, particularly after the initial neonatal period. We have previously shown that female 16-year-old children of mothers with ICP had increased waist and hip girth and lowered HDL-cholesterol, while males had an increased BMI and raised fasting insulin, relative to controls [5]. To explore this further, we used a mouse model of maternal CA-feeding to mimic the hypercholanemia seen in ICP [18]. Offspring in this model are predisposed to metabolic disease; 18-week-old female offspring of CA-fed mothers challenged with WD had significantly increased body weight, impaired glucose tolerance, and increased serum cholesterol and LDL cholesterol compared with controls. Furthermore, they had elevated hepatic cholesterol and free fatty acids, and hepatic steatosis [5]. The phenotype of male offspring was considerably milder, with only significantly increased liver and adipose tissue weight in WD-fed males of CA-fed mothers compared to those from NC-fed mothers [5]. Maternal CA-feeding impacted the epigenome of the offspring, but the precise mechanism of how the epigenetic changes can alter the offspring metabolism is yet to be defined [5]. In the current study, we found that maternal hypercholanemia also impacts the adult female offspring metabolome and microbiome, when challenged with WD.

WD-feeding in offspring of NC-fed dams tended to shift composition of the bile acid pool to favor primary bile acids and tauro-conjugated species, consistent with human data [28] and potentially indicative of decreased bacterial modification as often described in obesity. In female offspring exposed to maternal hypercholanemia, WD-feeding resulted in changes in the cecal bile acid profile, with specifically decreased relative CA and DHCA levels, a lower prevalence of primary bile acids, and decreased tauro-conjugation rates when compared to those from NC-fed mothers given the same obesogenic diet. Changes in cecal bile acid composition were less prevalent in females who were fed NC, from which we conclude that the obesogenic diet unmasks this programmed difference in bile acid metabolism. We hypothesized that altered cecal bile acid homeostasis could be due to changes in the offspring gut microbiome, leading to more de-conjugation and conversion of primary to secondary bile acids in the intestine. Accordingly, we found that WD challenge caused a separation in cecal bacterial communities between those born from NC- or CA-fed mothers, which was not seen in NC-fed offspring. Despite decreased levels of cecal CA and HDCA levels in female offspring, no significant changes were identified in individual genera known to metabolize bile acids in female offspring, such as *Lactobacillus*, *Bacteroides*, and *Clostridium*. However, it is plausible that the overall microbiotal capacity to modify intestinal bile acids was reduced in the NC WD offspring, consistent with a recent study in pediatric inflammatory bowel disease patients, which demonstrated that community-level microbial composition determined the potential for bile acid deconjugation and biotransformation in the intestine, rather than differences in individual genera between groups [36]. The trending increase in the Shannon index in CA-exposed offspring (consistent with increased diversity in CA-fed mothers [12]) shows more subtle bacterial changes may be contributory. Higher diversity is often associated with metabolic benefit, yet CA-WD females showed a more severe metabolic phenotype in our previous work [5]. These alterations may have been compensatory at first, but maladaptive once offspring were exposed to a Western diet.

Given that changes are evident in adult offspring at 18 weeks old, this led us to conclude that microbiota are likely also altered in the neonatal and infant period, considering that early microbial exposure is thought to define succession of bacteria which leads to the adult microbiota [10]. A recent study has reported changes in the microbiota of neonatal rats from CA-fed mothers [37]. The authors found no significant difference in alpha diversity, but they did see a difference in beta diversity in cecal contents of 7-day-old offspring by maternal CA-feeding. Our study shows that changes in the microbiota can persist into adulthood, presumably contributed to by altered vertical transmission from mother to offspring, which is plausible considering that the maternal gut microbiota is altered in pregnancy and ICP [12–15].

A limitation of our study is that only female offspring were included. We chose to focus on female offspring because the metabolic and hepatic phenotype following maternal CA-feeding was stronger in this sex [5]. Follow-up studies should include offspring of both sexes, especially because sexual dimorphism has been shown in response to prenatal exposure to maternal hypercholanemia [5] as well as in gut microbiota responses to dietary challenges [38]. Indeed, a recent study in mice showed that exposure to maternal high fat/high sucrose diet affected offspring gut microbiota and bile acid homeostasis differently, with males demonstrating decreased bacterial diversity and decreased *Bifidobacterium* spp. abundance and increased ratio of primary to secondary bile acids; while females displayed decreased *Eubacterium* spp. abundance [38]. In addition, this study has focused on the cecal contents, which show proportionately more secondary and deconjugated bile acids resulting from bacterial activity than the small intestine, and have a similar bile acid profile to the colon [39]. However, the gut microbiota influences the BA composition across the whole enterohepatic circulation [39], therefore further work is needed to evaluate bile acid composition in other compartments of the intestine and other tissues [39].

The murine model of CA feeding to mimic the hypercholanemia seen in ICP has its limitations when studying the maternal intestinal environment, but in terms of offspring bile acid exposure it provides similar changes in maternal serum bile acids to ICP, through which we postulate some programming impacts may occur. Accepting that acquisition of the initial neonatal microbiota does occur at birth and is contributed to by maternal microbiota changes, this is a limitation of this mouse model. Similarly, the impact of the paternal intestinal microbiotal composition has not been studied in this model, which is particularly relevant where coprophagy and horizontal transfer of paternal to maternal microbiota may occur. However, data from human cecal studies (maternal or neonatal) are not currently available as no acceptable sampling methodology has been reported in these populations, therefore murine studies are essential for furthering our understanding. While we have shown that CA exposure influences the effects of WD on female offspring, the mechanism remains unclear; future studies could address this by examining expression of bile acid transporters and homeostasis targets in the gut. Lastly, our microbiota data are preliminary and warrant further investigation. The work by Lin and co-workers reporting microbiome changes in newborn rats of cholestatic mothers [37] sparked our interest in revisiting historic data using old methods. Any method to characterize the microbiota is prone to contamination, a well-known challenge of microbiotal work. Our findings should thus be confirmed in other models using more recent methods of microbial investigation, and metabonomic results compared with larger group numbers to reduce the risk of model over-fitting.

Despite these limitations, this study shows that maternal exposure to CA while pregnant can impact the cecal metabolome and microbiota of adult offspring when challenged with an obesogenic diet, providing further evidence that exposure *in utero* to hypercholanemia can program long lasting changes to metabolism. ICP is a relatively common gestational disorder, affecting up to 1 in 20 pregnancies in some countries [2]. Given that we have previously demonstrated a predisposition of ICP offspring to obesity, dyslipidemia and insulin resistance, the microbiota could offer a potential route for therapeutic intervention to improve the cardiometabolic health of the children of affected women. Early studies investigating pre- and postnatal administration of probiotics have already been carried out for mothers with gestational diabetes mellitus or at risk of having children with allergic disease [40]. This study provides evidence supporting the evaluation of the potential of similar approaches to modify the gut microbiota and metabolome in children of pregnancies affected by ICP.

## Data Availability

1) The datasets presented in this study can be found in online repositories. The names of the repository/repositories and accession number(s) can be found below: https://info.figshare.com/, https://figshare.com/s/961c216fe5609932f483, https://www.ebi.ac.uk/ena, PRJEB89954 and 2) The metabolic profiling and rRNA data that support the findings of this study are openly available in FigShare at https://figshare.com/s/961c216fe5609932f483 [private sharing link] and at https://doi.org/10.5281/zenodo.17639265, respectively. The information will be publicly available at the time of publication.

## Footnotes

1
http://www.hmdb.ca/

2
http://www.genome.jp/kegg/ligand.html

3
https://www.lipidmaps.org/

4
http://metlin.scripps.edu/
