# Supplementary material for: Maternal diet-induced hypercholanemia alters gut microbiota and metabolome in adult female Western diet-fed offspring
Source: Exp Biol Med (Maywood). 2026 Jan 30;251:10810. doi: 10.3389/ebm.2026.10810 (PMC12902774; doi:10.3389/ebm.2026.10810)
Supplement: Supplementary file 1 [file Table1.docx]

**Supplementary Table S1 Bile acid profile of maternal cecal content**

|  |  | **Pregnant NC vs**  **non-pregnant NC** | | **Non-pregnant CA vs**  **non-pregnant NC** | | **Pregnant CA**  **vs pregnant NC** | |
| --- | --- | --- | --- | --- | --- | --- | --- |
| Metabolite | **Abbreviation** | **Fold change** | ***p*-value** | **Fold change** | ***p*-value** | **Fold change** | ***p*-value** |
| Cholic acid | CA | 3.39 | NS | 2.55 | NS | -1.15 | NS |
| Taurocholic acid | TCA | 3.73 | NS | 1.98 | NS | -1.51 | NS |
| β-Muricholic acid | β-MCA | -1.06 | NS | -3.95 | **0.02** | -3.08 | NS |
| ω-Muricholic acid | ω-MCA | 1.10 | NS | -2.57 | **0.03** | -5.39 | **0.02** |
| Tauro-Muricholic acids | TMCAs | 2.50 | NS | -6.62 | **0.03** | -26.24 | NS |
| Deoxycholic acid | DCA | -1.14 | NS | 2.70 | **<0.005** | 3.28 | **0.02** |
| 5-β-cholanic acid 3β,12a,diol | iso-DCA | -1.03 | NS | 2.26 | **<0.005** | 2.53 | NS |
| Lithocholic acid | LCA | -1.80 | **0.02** | -19.20 | **<0.001** | -7.88 | **0.02** |
| Hyodeoxycholic acid | HDCA | -6.21 | **0.02** | -8.07 | **0.01** | -2.42 | NS |
| Dehydrocholic acid | DHCA | 3.86 | NS | 2.75 | NS | -1.48 | NS |
| BA-Sulfated of m/z 391.3 (01) |  | 1.36 | NS | 387.65 | **<0.001** | 293.96 | **0.01** |
| BA-Sulfated of m/z 391.3 (02) |  | 1.13 | NS | 265.00 | **0.01** | 186.03 | **0.02** |
| BA-Sulfated of m/z 407.3 (01) |  | 1.35 | NS | 31.53 | **<0.005** | 17.49 | **0.01** |
| BA-Sulfated of m/z 407.3 (02) |  | 1.85 | NS | 115.65 | **<0.005** | 87.86 | **<0.005** |
| BA-Sulfated of m/z 407.3 (02) |  | -1.64 | NS | 1.16 | NS | 2.23 | NS |
| BA-Sulfated of m/z 407.3 (03) |  | -1.08 | NS | 38.24 | NS | 86.40 | **0.03** |

Table showing individual bile acid species present in the cecal content determined by bile acid profiling UHPLC-MS in non-pregnant and pregnant female mice fed either normal chow diet (NC) or cholic acid (CA)-supplemented diet. Significance determined by Student’s t-test following Pareto scaling and model fitting with SIMCA. Significant changes (*p*<0.05) are shown in bold. Fold change between pregnancy or dietary groups as shown. n = 4-6 per group. NS, not significant. BA, bile acids.
