# Supplementary material for: Maternal diet-induced hypercholanemia alters gut microbiota and metabolome in adult female Western diet-fed offspring
Source: Exp Biol Med (Maywood). 2026 Jan 30;251:10810. doi: 10.3389/ebm.2026.10810 (PMC12902774; doi:10.3389/ebm.2026.10810)
Supplement: Supplementary file 2 [file Table2.docx]

**Supplementary Table S2 Effect of maternal diet on bile acid species in offspring cecal content**

|  |  | **CA NC vs NC NC** | | **CA WD vs NC WD** | |
| --- | --- | --- | --- | --- | --- |
| Metabolite | **Abbreviation** | **Fold change** | **p-value** | **Fold change** | **p-value** |
| Cholic acid | CA | -1.07 | NS | -2.30 | **0.02** |
| Taurocholic acid | TCA | -1.43 | NS | -2.93 | NS |
| β-Muricholic acid | β-MCA | -1.23 | NS | -1.75 | NS |
| ω-Muricholic acid | ω-MCA | -1.21 | NS | 1.05 | NS |
| Tauro-Muricholic acids | TMCAs | -1.48 | NS | -1.67 | NS |
| Deoxycholic acid | DCA | -1.49 | NS | 1.03 | NS |
| 5-β-cholanic acid 3β,12a,diol | iso-DCA | -1.62 | NS | -1.26 | NS |
| Lithocholic acid | LCA | -1.14 | NS | 1.23 | NS |
| Hyodeoxycholic acid | HDCA | -1.30 | NS | -1.01 | NS |
| Dehydrocholic acid | DHCA | -1.78 | NS | -2.59 | **0.03** |
| BA-Sulfated of m/z 391.3 (01) |  | -1.30 | NS | -1.02 | NS |
| BA-Sulfated of m/z 391.3 (02) |  | 1.97 | NS | 1.27 | NS |
| BA-Sulfated of m/z 407.3 (01) |  | -1.05 | NS | 1.25 | NS |
| BA-Sulfated of m/z 407.3 (02) |  | -1.41 | NS | -1.79 | NS |
| BA-Sulfated of m/z 407.3 (02) |  | 1.12 | NS | -1.33 | NS |
| BA-Sulfated of m/z 407.3 (03) |  | 1.58 | NS | -1.46 | NS |

Individual bile acid species present in the cecal content of female offspring from mothers fed normal chow (NC) or cholic acid (CA)-supplemented diet and challenged with Western diet (WD) or control (NC), determined by bile acid profiling UHPLC-MS/MS. Fold change between dietary groups shown. Significance determined by Student’s t-test following Pareto scaling and model fitting with SIMCA. Significant changes (*p*<0.05) are shown in bold. NS, not significant. Females, n = 6 per group. BA, bile acids.
